# Assessing anxiety among adolescents in Hong Kong: psychometric properties and validity of the Generalised Anxiety Disorder-7 (GAD-7) in an epidemiological community sample

**DOI:** 10.1186/s12888-022-04329-9

**Published:** 2022-11-14

**Authors:** Hang Ip, Yi Nam Suen, Christy Lai Ming Hui, Stephanie Ming Yin Wong, Sherry Kit Wa Chan, Edwin Ho Ming Lee, Michael Tak Hing Wong, Eric Yu Hai Chen

**Affiliations:** 1 Department of Psychiatry, The University of Hong Kong, Rm 222, New Clinical Building, Queen Mary Hospital,102 Pokfulam Road, Hong Kong, Hong Kong; 2 State Key Laboratory of Brain and Cognitive Sciences, The University of Hong Kong, Hong Kong, Hong Kong

**Keywords:** Anxiety, GAD-7, validity, Rasch Model

## Abstract

**Background:**

The development of a valid and simple-to-use self-administered tool in Asian adolescents for clinical screening and intervention remains limited. The present study assessed the psychometric characteristics and validity of the Generalised Anxiety Disorder Scale-7 (GAD-7) among adolescents in Hong Kong.

**Methods:**

Epidemiological data from 3,261 Hong Kong adolescents aged 15 - 24 years were analysed for the construct validity, criterion validity, concurrent validity, and Rasch Model. All analyses were age- and gender-weighted according to the distributions of Hong Kong’s general population.

**Results:**

The GAD-7 showed high internal consistency and strong fit to the one-factor structure. The best cut-off value was set at 7 or more. Regression models found that the total scores of the scale were positively associated with symptoms of depression and hypomania, schizotypal personality and alcohol dependence. Rasch model analysis found that the separation index was 2.18 and 16.51 for the respondents and items, respectively and all residual pairs had small correlation coefficients (i.e., < 0.3).

**Conclusions:**

All psychometric findings presented in this study support the use of the GAD-7 as a legitimate measure of anxiety severity. A cut-off score of 7 should indicate a potential diagnosable condition in Asian adolescents, which requires our attention but should not be used as a formal diagnostic screening tool. The findings revealed the local dependence of the items of the GAD-7 and that the scale can separate respondents into at least two groups and items into numerous groups according to the separation index.

**Supplementary Information:**

The online version contains supplementary material available at 10.1186/s12888-022-04329-9.

## Introduction

Anxiety is one of the most common and profound mental illnesses across the world, impacting one-fifth of all young people at some point in their lives [1]. It has a negative impact on the overall health, functioning, well-being, quality of life and interpersonal relationships of affected individuals [2–4], with high comorbidity with many other physical and psychiatric disorders, such as cardiovascular disorders, depression, mania, panic disorders, etc [5–7]. Even modest anxiety should not be disregarded as innocuous, since those with unrecognised anxiety experienced equivalent or even worse declines in functioning and well-being [8]. There is also growing evidence that the overall prevalence of anxiety among young people has been progressively rising over the last decade [9, 10]. Given that adolescence and early adulthood are the prime years of psychosocial development [11], the long-term burden imposed on patients and society is innumerable [12].

All of these findings have underlined the significance of a low-cost, simple-to-implement and still reliable screening tool for generalised anxiety disorder. The Generalised Anxiety Disorder–7 items (GAD-7) scale is one of the most widely used and well-researched self-report scales [13]. Prior studies on the psychometric properties of the GAD-7 have demonstrated its broad applicability in a variety of age groups, cultures, and settings [14–16]. Several large-scale studies and reviews have previously revealed that this scale has a high level of internal consistency, sensitivity, and specificity [17–19]. Nonetheless, it should be highlighted that the validation of the GAD-7 in a large epidemiological sample of young people is still absent, not to mention in Asia.

According to the definition largely adopted by international organisations, 15 to 24 year-olds are considered “youths” who are capable of making the transition from dependence to independence [20]. Due to the peculiarity of this life period, particular care and attention are required to meet their demands. Anxiety disorders often manifest between the ages of 17 and 25 [21], highlighting the necessity of validating the GAD-7 in a youth population. This alarming fact about youth anxiety may be further complicated by greater cognitive symptoms of anxiety among young adults [22], its strong catalyst effect on depression [23], youths’ underdeveloped emotion regulation efficacy [24], and less perceived control and more anxiety-related worries in this age group [25].

Several studies have shown preliminary evidence for the application of the GAD-7 in Asia. A Korean study found that the GAD-7 has a unidimensional structure and high internal consistency in a sample of university students [26]. Another study found that the GAD-7 had a strong convergent and discriminant connection with factors including rumination, posttraumatic stress disorders, and perceived social support among Filipino migrant domestic workers [27]. These studies, however, only tested the applicability of the GAD-7 in a subset of adult samples, which may limit the generalisability of these results in the youth population. Despite the fact that the psychometric properties of the GAD-7 have already been tested in a large sample of Chinese adolescents [28], given the cultural differences between Hong Kong and the mainland due to the interweaving of historical and societal factors, discrepancies in item comprehension require further investigation.

The comorbidity of anxiety with other psychiatric conditions, particularly mood disorders [29], has been well established. Nonetheless, very little research has been done on the other so-called secondary symptoms, such as insomnia and alcohol dependency. It was found that some patients or practitioners may simply miss or underreport these “trivial” issues [30]. A recent study reported that although many people suffer from insomnia, which is a strong moderator of anxiety and functional impairment, neither complaints from patients nor diagnoses by clinicians were reported [31–33]. The findings on the relationship between anxiety and alcohol use disorders, hypomania, and schizotypal personality traits, on the other hand, are sporadic or mixed [34, 35]. To obtain a full picture of the scale’s convergent and divergent validity, it is indispensable to look at more of these areas of study.

While GAD-7 is a brief and widely used screening tool for GAD, establishing a culturally relevant cut-off would be critical for identifying individuals at increased risk. While a cut-off of 10 is commonly used, this cut-off still results in varying sensitivity and specificity across samples, settings and regions. For example, the optimal cut-off values reported in western studies involving primarily adult patient samples typically ranged between 10 and 12 [36–38], whereas those reported in Asian regions were typically between 5 and 7 [39–41]. Additional statistical adaptations of the scale are necessary for more precise diagnosis and intervention, particularly in regions such as Hong Kong, where there is a severe shortage of well-trained mental health professionals [42].

Since the GAD-7 has not been validated among an epidemiological sample of youth aged 15 to 24, the current study will assess the reliability and diagnostic validity of the Chinese-language version of the GAD-7 using the Composite International Diagnostic Interview (CIDI) as the gold standard. The GAD-7 scores for each demographic subgroup were then compared. The GAD-7 scale’s reliability, criterion validity, construct validity, concurrent validity, and item performance were assessed.

## Materials & Methods

### Participants

This study sample included 3,261 youths aged 15 to 24 who were recruited between 17 May 2019 and 2 April 2022 as part of a large-scale, ongoing epidemiological study in Hong Kong. The study adopted a random sampling method in which participants were invited through mail using the address lists given by the Census and Statistical Department of the Hong Kong SAR Government. The addresses were prestratified by geographical location and type of housing quarters. This study does not have particular exclusion criteria as long as the participant resided in the household that received our invitation, was in the appropriate age range, and could provide written consent (or parental consent if the participant is aged 15 - 17) for participation. All procedures were carried out in accordance with the protocol approved by the ethics committee.

### Data collection

Participants who met the eligibility criteria and received an invitation letter might indicate their preference for participation through a registration website or a telephone hotline. The participants with confirmed eligibility were interviewed in person by trained researchers about the interviewer-rated measures, which were supplemented by several self-administered measures on sociodemographic profile, health-related lifestyle, psychopathological experiences, childhood adversities, life stressors, psychosocial functioning and service utilisation. The present study comprised data from 3,261 participants who received diagnostic interviews for GAD and provided no missing data for the measures of interest.

### Measures

#### Generalised Anxiety Disorder-7 (GAD-7)

Participants’ anxiety symptoms were evaluated by the GAD-7, which is a brief self-administered rating scale that assesses the severity of anxiety symptoms in the past 2 weeks [13]. The scale consists of seven items that are statements about worry or somatic symptoms and are rated on a four-point Likert scale ranging from 0 (not at all) to 3 (nearly every day), for a total score of 0 to 21. A higher score indicates that anxiety symptoms are more severe. The author suggested that, using interviews from mental health professionals as the gold standard, a cut-off of 10 could yield a sensitivity of 89% and a specificity of 82% [13]. With a Cronbach’s α coefficient of .92, the GAD-7 has been validated as a reliable measurement. Additionally, the scale demonstrated strong convergent (Beck Anxiety Inventory: *r* =.72; Symptom Checklist-90: *r* = 0.74) and divergent validity in patient samples collected from primary care sites (i.e., The GAD-7 scores were significantly higher in patients diagnosed with anxiety than in those who were not) [16]. The current study adapted the Chinese version of the scale [41].

#### *The World Health Organisation World Mental Health Composite International Diagnostic Interview - Screening Scales (CIDI-SC*)

The CIDI-SC is a reliable, comprehensive and widely used interviewer-administered diagnostic interview assessing psychiatric disorders in epidemiological and clinical studies [43]. As the GAD-7 assesses anxiety symptoms in the past two weeks, only items pertaining to the 30-day GAD were used in this study. The CIDI-SC GAD contains 12 items measuring the extent of anxiety symptoms in the past 30 days on a 5-point Likert scale from 0 (not at all) to 5 (all or almost all the time) in accordance with the diagnostic criteria of ICD-10 and DSM-IV, followed by 2 questions that rule out comparable experiences caused by substance use or general medical condition. According to the CIDI diagnostic criteria, interviewees are considered positive for the 30-day GAD if they report significant anxiety-related symptoms and difficulty regulating excessive anxiety, as well as clinically significant discomfort with a series of events or activities or impairment that cannot be better explained by the physiological effects of a substance or another medical condition and another mental disorder.

### Other measures

#### Patient Health Questionnaire (PHQ-9)

The PHQ-9 was used to evaluate participant’s severity of depressive symptoms in the past two weeks [44]. The scale consists of nine items, each of which is rated on a four-point Likert scale ranging from 0 (not at all) to 3 (nearly every day), for a total score of from 0 to 27. Similar to the GAD-7, a cut-off of 10 could yield a sensitivity of 88% and a specificity of 88% when interviews with mental health professionals are used as the gold standard. Additionally, the PHQ-9 also demonstrated a high level of internal consistency (Cronbach’s *α* = .86 - .89) in clinical samples. It has a strongly positive correlation with overall mental health (*r* = .73) and a moderate-to-weak, positive correlation with general health perceptions (*r* = .55), functioning (social: *r* = .52; role: *r* = .43; and physical: *r* = .37), and bodily pain (*r* = .50) [44]. These findings substantiated both the convergent and divergent validity of this instrument. The Chinese version of the scale, which was previously validated by researchers in China, was used in the current study [45].

#### Hypomania Checklist 32 (HCL-32)

We also assessed participant’s lifetime experience of hypomania using the HCL-32 [46]. The scale consists of 32 questions that assess the presence of a range of symptoms such as inflated self-esteem, reduced needs for rest or sleep and heightened communication or urge to keep talking, etc. Respondents are asked to focus on a particular moment of “high mood” and then indicate if certain thoughts, feelings, and actions were present during this period. The scale also contains 8 items pertaining to the severity and functional impact that are not included in the total score. The total score of this scale is determined by the number of positive answers to the 32 questions investigating specific symptoms. Several studies have reported on its factor structure, which is “active/elated” and “risk-taking/irritable.” This scale had strong internal consistency (α = .82) and sensitivity (80%), but its specificity (51%) was far below standard. The scale was previously in youth sample in German [47] but not in Chinese, while validation in Chinese was found only among clinical sample [48].

#### Alcohol Use Disorders Identification Test (AUDIT)

The AUDIT is a screening tool with 10 questions that assesses hazardous alcohol dependency and its associated harmful consequences [49], and the current study used a 12-month time frame. Each item is rated on a five-point Likert scale from 0 to 4, with total scores ranging between 0 to 40. Higher scores indicate a higher likelihood of alcohol-related problems. The first three questions assess alcohol consumption, the 4th to 6th measure alcohol dependency, and the last four assess alcohol-related problems. The scale was validated in a study including patients from six countries [49]. The Cronbach’s *α* coefficient (.93), sensitivity (87% - 96%) and specificity (81% - 98%) of this scale reported in previous studies were excellent [49]. The scale was previously validated in a youth sample in Western countries (e.g., Liskola et al .[50]) but not in a Chinese population.

#### Schizotypal Personality Questionnaire Brief (SPQ-B)

The SPQ-B is a self-report questionnaire with 22 items that screens for schizotypal personality disorder [51]. This scale is further subdivided into three subscales, each of which containing statements reflecting the cognitive-perceptual and interpersonal deficits, as well as disorganisation, that are frequent in schizotypal personality. The total score is determined by the number of “agree” responses chosen. At the cut-off of 17, the Chinese version of the scale demonstrated a promising internal consistency of *α* = .76, as well as a good sensitivity of 80% and specificity of 85.9% [52]. The SPQ-B had a high correlation with the Millon Adolescent Clinical Inventory, a diagnostic assessment of adolescents’ mental health, and a moderate correlation with the Adolescent Dissociative Experience Scale [53], yet validation of convergence or divergence against anxiety is still lacking.

#### Insomnia Severity Index (ISI)

The ISI is a brief measure for evaluating the severity and impacts of insomnia [54]. Participants are required to rate on three items based on the severity of 1) difficulty falling asleep, 2) difficulty staying asleep, and 3) problem waking too early. This section is followed by four further questions that record the level of dissatisfaction, impairment, distress and interference caused by the respective sleep problems. All seven questions are rated on a Five-point Likert scale from 0 to 4. This scale is totalled up to 28. This scale possesses a satisfactory sensitivity of 86.1% and specificity of 87.7%, and its Cronbach’s *α* coefficient of this scale was excellent (α = .90 - .91 )[55]. The scale was previously validated in Chinese adolescents [56].

### Statistical analysis

Data on the participant’s sex, age, psychiatric history, and educational status were collected. Normality tests included Kolmogorov–Smirnov, Shapiro–Wilk, and Q–Q plots. The distribution of GAD-7 scores did not meet the normality assumption for parametric testing. The Mann–Whitney U test was used to compare GAD-7 scores between sexes, participants with and without psychiatric histories, and educational levels, while the Kruskal-Wallis test was used to compare GAD-7 scores between age groups.

The validity of the GAD-7 was evaluated using two approaches: (1) classical test theory (CTT) and (2) item response theory (IRT). The CCT examined construct, criterion, and concurrent validity, while the IRT examined the Rasch Rating Scale model for polytomous data. Since the GAD-7 is a unidimensional scale, no discriminant validity was tested. All statistical analyses were performed using IBM SPSS Statistics 28.0 and WINSTEPS 5.2.2.0.

#### Construct validity

Cronbach’s alpha was computed to assess the internal consistency of the GAD-7. A value greater than .70 as the standard index of acceptable reliability was adopted [57]. Before examining the factor structure of the scale, the study sample adequacy and suitability for scale reduction for factor analysis were then tested using the Kaiser-Meyor-Olkin (KMO) test and Bartlett’s test of sphericity. Exploratory factor analysis (EFA) was then conducted to explore the underlying factor structure of the GAD-7. To evaluate the goodness of fit of the model, four indexes, including the comparative fit index (CFI), Tucker–Lewis index (TLI), root mean square error of approximation (RMSEA), and standardised root-mean-square residual (SRMR), were used. Cut-off values for CFI > 0.90 [58], TLI > 0.90 [59], RMSEA < 0.08 [60], and SRMR < 0.08 [61] suggest a satisfactory fit of the model. The scree plot was generated to assist in identifying the number of factors, with an eigenvalue > 1 and factor loadings ≥ 0.30 chosen.

#### Criterion validity

By conducting a receiver operating curve (ROC) analysis with the Composite International Diagnostic Interview (CIDI) as the gold diagnostic benchmark, the sensitivity, specificity, positive (PPVs) and negative predictive values (NPVs), positive (LR+) and negative likelihood ratio (LR-), and the area under the curve (AUC) were measured to evaluate the criterion validity of the GAD-7. The Youden index was computed by subtracting 1 from the sum of the sensitivity and specificity of the test (i.e., (sensitivity + specificity) - 1 )[62] . It is regarded as an objective measurement of the maximum scale performance at different cut-offs, and the optimal cut-off score was determined by the highest Youden index.

#### Concurrent validity

A multiple linear regression was used to examine whether the severity of anxiety symptoms measured by the GAD-7 was associated with other psychiatric symptoms, such as depression, hypomania, schizotypal personality traits, alcohol use and sleep problems. The GAD-7 total score was entered into the regression model as an independent variable, and participants’ sociodemographics and past psychiatric history were entered as the control variables.

#### Rasch Rating Scale model

The overall quality of the GAD-7 was also evaluated using Rasch analysis based on three domains of measures: (1) reliability and separation for items and respondents, (2) item fit statistics, (3) rating scale diagnostics, and (4) unidimensionality and local dependence [63]. A reliability score, similar to Cronbach’s alpha, if it is greater than .80, is regarded as a satisfactory confidence level of the measure. A separation index greater than 2 indicates that the scale is sufficiently sensitive to separate the item responses or respondents into at least two groups, which is regarded as preferred [64]. The item fit statistics are presented in terms of the logit of difficulty, standard errors, infit and outfit mean square (MnSQ) and standard scores (Zstd). Generally, an infit and outfit mean square closer to 1 indicates less distortion of the measurement system. The acceptable range for the infit index is from 0.6 to 1.4 and that for the outfix index is from 0.5 to 1.7 [65]. The infit is more sensitive to abnormal patterns of observations by persons on items that are roughly targeted on them (and vice versa). The outfit is more sensitive to abnormal observations by persons on items that are relatively easy or very hard for them (and vice versa). The rating scale diagnostics were used to evaluate how well the categories that make up the response set functioned to create an interpretable measure. For each category, we examined the shape of the distribution and the number of endorsements the response received. The unidimensionality of the GAD-7 was examined by conducting principal component analysis of the residuals after fitting the Rasch model, as implemented in WINSTEPS software. An eigenvalue <2 of the unexplained variance in each component indicates the unidimensionality of the scale. The local independence (which means the response to one item has no influence on the response to another) of the GAD-7 was examined by examining the correlation between item residuals after partialling out the Rasch dimension.

## Results

As Table 1 shows, slightly more of the 3261 participants were male, and the majority were aged 18 or older, were students and had no psychiatric history. The mean GAD-7 score was 4.71 (SD = 4.52), and 1.6% of the participants met the criteria for GAD based on the CIDI-SC. GAD scores were higher in females, older age, nonstudents, participants with a psychiatric history and those who tested positive on the CIDI-SC 30-day GAD (all p-values ≤ .01).

**Table 1:** Socio-demographics, GAD-7 scores and 30-day GAD of the study sample (N = 3,261)

Characteristics	Total Sample (N = 3,261)		GAD-7 scores	***P***-value	Effect size
	n	%	Mean ± SD		
Sex					
Male	1638	52.0	4.24 ± 4.42	<0.001	*r* = −0.129
Female	1623	49.8	5.19 ± 4.57		
Age					
15-17	782	24.0	4.37 ± 4.34	0.011	${E}_R^2$ = 0.002
18-21	1215	37.2	4.65 ± 4.55		
22-24	1264	38.8	4.97 ± 4.58		
Currently in Education					
Yes	2432	74.6	4.52 ± 4.37	0.001	*r* = −0.057
No	829	25.4	5.27 ± 4.90		
Lifetime psychiatric history					
Yes	325	10.0	7.39 ± 5.88	<0.001	*r* = −0.167
No	2936	90.0	4.41 ± 4.24		
GAD-7 total score (mean, SD)	n/a	n/a	4.71 ± 4.52	n/a	n/a
30-day GAD					
Yes	51	1.6	12.21 ± 5.86	<0.001	*r* = −0.156
No	3210	98.4	4.59 ± 4.39		

*r* = correlation coefficient ranging from –1.00 to 1.00; $\mathrm E_{\mathrm R}^2$= epsilon-squared estimate of effect size, the coefficient assumes the value from 0 (indicating no relationship) to 1 (indicating a perfect relationship)

### Construct validity

Cronbach’s *α* coefficient of .922 supported the excellent internal consistency of the GAD-7, and this coefficient would drop if any items were deleted (Table 2). Both the KMO index (0.931) and the significant result of Bartlett’s test of sphericity (*p* < .001) indicated that the sample size was adequate and fitted for the subsequent EFA test. Eigenvalues and the scree plot suggested a unidimensional model with very good model fit (CFI: 0.983; TLI = 0.975; RMSEA = 0.056 (90% CI: 0.049 - 0.064); SRMR = 0.018).

**Table 2:** Corrected item-total correlations, Cronbach’s α after item deletion* and factor loading of the GAD-7

Items	Corrected item-total correlation	Cronbach’s ***α*** if item deleted	Factor Loading
1. Feeling nervous, anxious, or on edge	0.764	0.909	0.799
2. Not being able to stop or control worrying	0.819	0.903	0.866
3. Worrying too much about different things	0.793	0.906	0.839
4. Trouble relaxing	0.767	0.909	0.806
5. Being so restless that it’s hard to sit still	0.771	0.909	0.800
6. Becoming easily annoyed or irritable	0.706	0.915	0.732
7. Feeling afraid as if something awful might happen	0.677	0.917	0.706

*The overall Cronbach’s alpha is .922

### Criterion validity

**Fig. 1:**
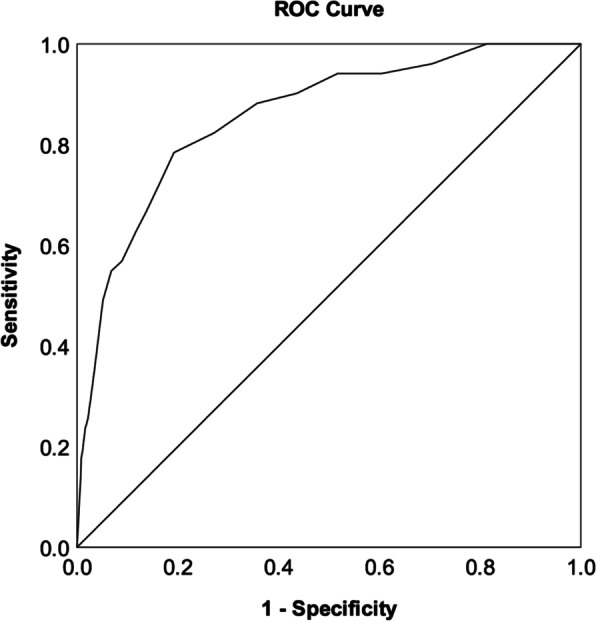
ROC curve of the GAD-7 based on the CIDI-GAD diagnostic outcome

The sensitivity, specificity, Youden index, PPVs, NPVs, LR+ and LR- at different cut-offs of the GAD-7 total scores were calculated. The results using the cut-off values from 5 to 10 all showed a Youden Index larger than 0.50 and thus are presented in Table 3. The specificity increased with a higher cut-off but at the expense of sensitivity. The optimal cut-off based on the current sample was 7, with a sensitivity of 79.2% and specificity of 80.9%, and PPV and NPV were 4.5% and 99.6%, respectively. The LR+ and LR- for the cut-off values of 5 - 10 increased from 2.47 to 5.36 and 0.18 to 0.42, respectively. The area under the ROC curve for the GAD-7 versus the CIDI 30-day GAD was 0.86 (S.E. = 0.027; 95% CI = 0.806 - 0.913), which was significant (Fig 1).

**Table 3:** Diagnostic efficiency of the GAD-7 with CIDI-GAD as a gold standard

Cut-off	Sensitivity	Specificity	YI	PPV (%)	NPV (%)	LR+	LR-
≥5	0.882	0.643	0.525	3.2	99.7	2.47	0.18
≥6	0.824	0.728	0.552	3.8	99.7	3.03	0.24
≥7	0.784	0.808	0.592	4.5	99.6	4.08	0.27
≥8	0.725	0.835	0.560	6.2	99.5	4.39	0.33
≥9	0.667	0.863	0.530	6.5	99.4	4.87	0.39
≥10	0.627	0.883	0.510	7.1	99.4	5.36	0.42

*YI* Youden Index, *PPV* Positive predictive value, *NPV* Negative predictive value, *LR+* Positive likelihood ratio, *LR-* Negative likelihood ratio

### Concurrent validity

The linear regression coefficients of GAD-7 regressing on other variables are listed in Table 4. The GAD-7 had the greatest significant association with the PHQ-9, followed by SPQB, HCL-32 and then AUDIT-12. No association between GAD-7 and ISI was observed.

**Table 4:** Regression model showing the association between GAD-7 and other psychiatric conditions

	Dependent variable				
	PHQ-9	HCL-32	SPQB	AUDIT	ISI
	***β*** (***t***)	***β*** (***t***)	***β*** (***t***)	***β*** (***t***)	***β*** (***t***)
**Independent variable**					
GAD-7	0.737 (61.659)***	0.109 (6.109)***	0.414 (25.409)***	0.061 (3.471)***	0.029 (1.639)
**Control variables**					
Female (ref.: male)	−0.009 (−0.727)	−0.065 (−3.721)***	−0.069 (−4.338)***	−0.125 (−7.318)***	−0.015 (−0.832)
Age^a^	−0.035 (−2.549)*	0.107 (5.242***)	−0.004 (−0.210)	0.211 (10.560)***	−0.036 (−1.761)
Student (ref.: non-student)	0.004 (0.290)	0.033 (1.603)	−0.004 (−0.210)	0.003 (0.132)	−0.009 (−0.451)
Had psychiatric history (ref.: no history)	0.056 (4.685)***	−0.047 (−2.683)**	0.052 (3.195)**	0.011 (0.637)	0.012 (0.667)
*R*^2^	0.560***	0.024***	0.182***	0.062***	0.003

*β* = Standardised regression coefficient, *t* = t-test value, *ref.* = Referent of a binary variable, ^a^The age variable was put into the regression model as an ordinal variable. Therefore the value of the regression coefficients indicate the points the dependent variable increase (or decrease) as the age group changes from a young group to an older group. ***p < .001, **p <.01, *p < .05

### Rasch Rating Scale Model

Both the items (>0.99) and respondents (0.83) demonstrated a good reliability level, which means that we have very good confidence about the measures of the items and the respondents. The separation index was 16.51 and 2.18 for the items and respondents, respectively, which means that the scale is able to differentiate the responses to more than 16 levels and respondents to more than 2 levels, based on its difficulty. Table 5 presents Rasch-based item statistics for each of the 7 items. The lower the item difficulty value, the higher the endorsement. The item rated highest (i.e., lowest logit of difficulty estimate) was item 1 (−0.88; “Feeling nervous, anxious, or on edge”), and the item rated lowest (i.e., highest logit of difficulty estimate) was item 7 (1.16; “Feeling afraid as if something awful might happen”). All items were within the range of reasonable fit (Infit mean square: 0.82 - 1.33; Outfit mean square: 0.76 - 1.26).

**Table 5:** Item fit statistics of the GAD-7 using the Rasch Rating Scale Models

	Logit of difficulty		Infit^a^		Outfit^b^		***ρ***^c^
Item	Estimate	S.E.	MnSQ	Zstd	MnSQ	Zstd	
1. Feeling nervous, anxious, or on edge	−0.88	0.04	0.83	−6.57	0.84	−5.96	.83***
2. Not being able to stop or control worrying	0.21	0.04	0.82	−6.92	0.76	−8.39	.85***
3. Worrying too much about different things	−0.37	0.04	0.89	−4.10	0.87	−4.69	.85***
4. Trouble relaxing	−0.56	0.04	1.01	0.45	1.00	−0.03	.83***
5. Being so restless that it’s hard to sit still	0.79	0.04	0.94	−2.24	0.87	−3.69	.81***
6. Becoming easily annoyed or irritable	−0.35	0.04	1.22	7.66	1.22	7.23	.78***
7. Feeling afraid as if something awful might happen	1.16	0.04	1.33	9.90	1.26	5.53	.74***

*S.E.* Standard error, *MnSQ* Mean square, *Zstd* Standardised score, *ρ* Spearman’s correlation coefficient, ^a^Infit mean square value should range from 0.6 to 1.4, closer to 1 indicates better fit; ^b^Outfit mean square value should range from 0.5 to 1.7, closer to 1 indicates better fit. ^c^Spearman’s correlation between item and total score

The rating scale diagnostics found that the distribution of the observed frequencies was positively skewed, with the majority of the total endorsement falling in the first and second category. As the average endorsements increase monotonically across the rating scale (see threshold in Table 6 and supplementary material S1 for graphic representation), collapsing categories were not needed. The raters needed 2.10 logits to go from “Not at all” at −4.22 to “Several days” at −2.12, 2.53 logits to go from “Several days” at −2.12 to “More than half the days” at 0.41, and 2.16 logits to go from “More than half the days” at 0.41 to “Near every day” at 2.57”. The findings suggested that it was easier for the raters to move from category 1 to 2 and 3 to 4 than 2 to 3. In addition, none of the infit (outfit) mean square measures exceeded the reasonable ranges, indicating that no noise was introduced into the measurement process; thus, we can conclude that the response set of the GAD-7 functioned well. After partialling out the Rasch dimension, the first component from the matrix of residuals revealed the unidimensionality of the scale (supplementary material S2), and no item residuals correlated with each other with a correlation coefficient greater than 0.3, suggesting the local dependence of the scale (supplementary material S3).

**Table 6:** Summary of *r*ating *s*cale *d*iagnostics

Category	Observed count (%)	Expected score measure	Average measure	Threshold	Infit MnSQ	Outfit MnSQ
0 = Not at all	11205 (49.1)	−4.22	−4.24	-	1.03	1.00
1 = Several days	8480 (37.1)	−2.12	−2.12	−3.43	0.94	0.90
2 = More than half the days	2338 (10.2)	0.41	0.60	0.48	0.94	0.94
3 = Nearly every day	804 (3.5)	2.57	2.33	2.95	1.27	1.35

*MnSQ* Mean square, *Zstd* Standardised scoreρ: Spearman’s correlation coefficient; ^a^Infit mean square value should range from 0.6 to 1.4, closer to 1 indicates better fit; ^b^Outfit mean square value should range from 0.5 to 1.7, closer to 1 indicates better fit. ^c^Spearman’s correlation between item and total score

## Discussion

The current study examined the validity of the Chinese version of the GAD-7 in detail using a representative epidemiological sample of adolescents aged 15 to 24 years in Hong Kong. The findings indicate that the GAD-7 is a valid and reliable tool for identifying youths with a probable GAD state in Hong Kong. Similar to previous research, analyses revealed the GAD-7’s unidimensional structure, local dependence and appropriate rating scale design. Additionally, we found a strong association between it and a variety of other psychopathological problems, including depression and hypomania, as well as schizotypal personality and alcohol consumption, but our data do not suggest an association with sleep quality.

A cut-off of 7 for the CIDI diagnostic interview yielded the highest Youden Index, with a sensitivity and specificity of 78.4% and 80.8%, respectively. That acceptable range for the Youden Index was between scores 5 and 10, which is comparable with previous studies in the general population using the CIDI as the gold standard and reported cut-off scores between 5 and 17 [36]. Our findings add to the body of knowledge by suggesting a cut-off score of 7 for a younger target group. After all, practitioners may adjust the cut-off value depending on psychometric features to meet their specific needs. While the extremely high NPV showed that this scale can properly identify negative cases, the very low PPV (6%) showed that it cannot accurately detect positive cases. The low 30-day prevalence of GAD (1.6%) determined by CIDI in our sample may explain this scenario (i.e., the prevalence is too low to be accurately detected). In addition, the strict criteria of the CIDI may lead to an underestimation of GAD prevalence [66]. Thus, the GAD-7 scores should be interpreted with caution since they can only evaluate symptom severity and not diagnose a disorder state.

Our data confirm that the GAD-7’s widespread use among young Chinese people is appropriate, as reflected by its high internal inconsistency. The study identified a one-factor model with a very excellent match between observed and predicted values. While bidimensional structure has been identified in other samples [67, 68], we utilised EFA to explore a possible bidimensional GAD-7 structure in our sample (supplementary material S4). We found that items 2 and 3 load on the same latent factor, but items 4 and 7 load on another. Item 1 showed higher loading on the first latent factor, whereas item 6 showed more loading on the second. The findings suggested that a bidimensional structure does not seem to be valid in our sample.

In line with previous studies, we found that women are often more worried than men. There were significant age differences, with older youth experiencing more anxiety overall. Participants who were students reported being less worried than nonstudents. It is speculated that these outcomes may reflect the fact that young people who should have graduated from high schools or universities may have greater in-adaptability and anxieties after a long period of highly structured school life [69]. The current study is perhaps one of the few studies to examine the association between anxiety and hypomania, which was relatively weak, while the majority of previous research focused on bipolar II disorder [70, 71]. Nonetheless, we should not overlook this weak association given the apparent progression from hypomania to bipolar disorder [72]. Our findings also suggest that evaluating schizotypal personality characteristics and alcohol use may help us better identify youths at risk for anxiety. The findings are in line with previous research that found that severely dependent adolescent male drinkers reported significantly higher levels of anxiety [73]. Besides, previous studies also found that approximately half of the adolescents with anxiety disorders reported to have others comorbid psychiatric conditions including depressive disorders, somatoform disorders and substance use disorders [74]. Meanwhile, in a recent Rome study, around one-sixth of the children and adolescents with attention deficit hyperactivity disorder (ADHD) were also diagnosed with GAD [75]. Future study investigating these comorbid conditions is therefore warranted.

One of the strengths of the study is its use of an epidemiological sample of Hong Kong adolescents, which offered support for the scale’s usage at the population level. The availability of an accessible and valid self test might increase youths’ motivation to monitor their mental health. In addition to multiple group comparisons, the GAD-7 was evaluated comprehensively for its validity. This research may be the first in Asian countries to provide sensitivity, specificity, and cut-off values for the GAD-7 in an exclusively adolescent sample, which may pave the way for future investigations. This study extended the scope of association research to include less commonly examined "secondary" symptoms. This should help us understand the GAD-7’s structure and characteristics, as well as help us construct a more complete clinical consideration.

Nonetheless, we recognise the limitations of the study. First, due to the cross-sectional design, this research was unable to investigate the GAD-7’s predictive validity or the degree to which its findings predict future measures across time. A longitudinal strategy may be used in the future to evaluate the correlation between the baseline GAD-7 and associated future outcomes. Second, for cost-effectiveness reasons, the present study’s diagnostic evaluation, the CIDI, was performed by a group of trained psychology graduates rather than clinicians. Nevertheless, the trainees were supervised closely by a team of psychiatrists. If financially feasible, follow-up research may include obtaining support from licenced practitioners. Last, young people may have difficulty concentrating properly on this sophisticated face-to-face interview after the self-administered questionnaires, which may skew the prevalence further [76].

## Conclusion

In summary, our findings from a large epidemiological sample in Hong Kong indicated that the GAD-7 is a reliable measure of young people’s current anxiety levels. It has a high degree of reliability, convergent validity, and a well-fitting unidimensional structure. The best cut-off value for this scale is 7, and it has a high sensitivity and specificity. The GAD-7 is an efficient, easy-to-use, and valid measure of anxiety severity that aids in subsequent clinical diagnosis.

## Supplementary Information

**Additional file 1.**

## Data Availability

Data available on request from the corresponding author.

## References

[1] Baxter AJ, Scott KM, Vos T, Whiteford HA (2013). Global prevalence of anxiety disorders: a systematic review and meta-regression. Psychological Medicine.

[2] Auerbach RP, Mortier P, Bruffaerts R, Alonso J, Benjet C, Cuijpers P, Demyttenaere K, Ebert DD, Green JG, Hasking P, Murray E, Nock MK, Pinder-Amaker S, Sampson NA, Stein DJ, Vilagut G, Zaslavsky AM, Kessler RC, Collaborators WW-I (2018). WHO World Mental Health Surveys International College Student Project: Prevalence and distribution of mental disorders. J Abnorm Psychol.

[3] Przeworski A, Newman MG, Pincus AL, Kasoff MB, Yamasaki AS, Castonguay LG, Berlin KS (2011). Interpersonal pathoplasticity in individuals with generalised anxiety disorder. J Abnorm Psychol.

[4] Stein MB, Heimberg RG (2004). Well-being and life satisfaction in generalised anxiety disorder: comparison to major depressive disorder in a community sample. J Affect Disord.

[5] Nutt D, Argyropoulos S, Hood S, Potokar J (2006). Generalised anxiety disorder: A comorbid disease. Eur Neuropsychopharmacol.

[6] Tully PJ, Cosh SM, Baune BT (2013). A review of the affects of worry and generalised anxiety disorder upon cardiovascular health and coronary heart disease. Psychol Health Med.

[7] Wittchen HU, Zhao S, Kessler RC, Eaton WW (1994). DSM-III-R generalised anxiety disorder in the National Comorbidity Survey. Arch Gen Psychiatry.

[8] Schonfeld WH, Verboncoeur CJ, Fifer SK, Lipschutz RC, Lubeck DP, Buesching DP (1997). The functioning and well-being of patients with unrecognised anxiety disorders and major depressive disorder. J Affect Disord..

[9] Goodwin RD, Weinberger AH, Kim JH, Wu M, Galea S (2020). Trends in anxiety among adults in the United States, 2008–2018: Rapid increases among young adults. J Psychiatric Res.

[10] Parodi KB, Holt MK, Green JG, Porche MV, Koenig B, Xuan Z (2022). Time trends and disparities in anxiety among adolescents, 2012–2018. Soc Psychiatry Psychiatr Epidemiol.

[11] Meeus W (2016). Adolescent psychosocial development: A review of longitudinal models and research. Dev Psychol.

[12] Wittchen H-U (2002). Generalised anxiety disorder: prevalence, burden, and cost to society. Depress Anxiety.

[13] Spitzer RL, Kroenke K, Williams JBW, Löwe B (2006). A brief measure for assessing generalised anxiety disorder: the GAD-7. Arch Intern Med.

[14] García-Campayo J, Zamorano E, Ruiz MA, Pardo A, Pérez-Páramo M, López-Gómez V, Freire O, Rejas J (2010). Cultural adaptation into Spanish of the generalised anxiety disorder-7 (GAD-7) scale as a screening tool. Health Qual Life Outcomes.

[15] Hinz A, Klein AM, Brähler E, Glaesmer H, Luck T, Riedel-Heller SG, Wirkner K, Hilbert A (2017). Psychometric evaluation of the Generalised Anxiety Disorder Screener GAD-7, based on a large German general population sample. J Affect Disord.

[16] Johnson SU, Ulvenes PG, Øktedalen T, Hoffart A. Psychometric Properties of the General Anxiety Disorder 7-Item (GAD-7) Scale in a Heterogeneous Psychiatric Sample. Front Psychol. 2019;10. 10.3389/fpsyg.2019.01713 PMC6691128 31447721

[17] Herr NR, Williams JW, Benjamin S, Mcduffie J (2014). Does this patient have generalised anxiety or panic disorder?: The Rational Clinical Examination systematic review. JAMA.

[18] Löwe B, Decker O, Müller S, Brähler E, Schellberg D, Herzog W, Herzberg PY (2008). Validation and standardisation of the Generalised Anxiety Disorder Screener (GAD-7) in the general population. Med Care.

[19] Plummer F, Manea L, Trepel D, Mcmillan D (2016). Screening for anxiety disorders with the GAD-7 and GAD-2: a systematic review and diagnostic metaanalysis. Gen Hosp Psychiatry.

[20] Bersaglio B, Enns C, Kepe T (2015). Youth under construction: The United Nations’ representations of youth in the global conversation on the post-2015 development agenda. Can J Dev Studies/Revue canadienne d’études du développement.

[21] Lijster JMD, Dierckx B, Utens EM, Verhulst FC, Zieldorff C, Dieleman GC, Legerstee JS (2017). The age of onset of anxiety disorders: a meta-analysis. Can J Psychiatry.

[22] Brenes GA, Knudson M, McCall WV, Williamson JD, Miller ME, Stanley MA (2008). Age and racial differences in the presentation and treatment of generalized anxiety disorder in primary care. J Anxiety Disord.

[23] Jacobson NC, Newman MG (2017). Anxiety and depression as bidirectional risk factors for one another: A meta-analysis of longitudinal studies. Psychol Bull.

[24] Young KS, Sandman CF, Craske MG (2019). Positive and negative emotion regulation in adolescence: links to anxiety and depression. Brain Sci.

[25] Gould CE, Edelstein BA (2010). Worry, emotion control, and anxiety control in older and young adults. J Anxiety Disord.

[26] Lee B, Kim YE (2019). The psychometric properties of the Generalised Anxiety Disorder scale (GAD-7) among Korean university students. Psyc Clin Psychopharmacol.

[27] Garabiles MR, Lao CK, Yip P, Chan EWW, Mordeno I, Hall BJ (2020). Psychometric Validation of PHQ-9 and GAD-7 in Filipino Migrant Domestic Workers in Macao (SAR). China J Pers Assess.

[28] Sun J, Liang K, Chi X, Chen S (2021). Psychometric Properties of the Generalised Anxiety Disorder Scale-7 Item (GAD-7) in a Large Sample of Chinese Adolescents. Healthcare (Basel).

[29] Kessler RC, Petukhova M, Sampson NA, Zaslavsky AM, Wittchen H-U (2012). Twelve-month and lifetime prevalence and lifetime morbid risk of anxiety and mood disorders in the United States. Int J Methods Psychiatr Res.

[30] Leger D, Poursain B (2005). An international survey of insomnia: underrecognition and undertreatment of a polysymptomatic condition. Curr Med Res Opin.

[31] Almeneessier AS, Alamri BN, Alzahrani FR, Sharif MM, Pandi-Perumal SR, Bahammam AS (2018). Insomnia in primary care settings: Still overlooked and undertreated?. J Nat Sci Med.

[32] Neckelmann D, Mykletun A, Dahl AA (2007). Chronic Insomnia as a Risk Factor for Developing Anxiety and Depression. Sleep.

[33] Soehner AM, Harvey AG (2012). Prevalence and functional consequences of severe insomnia symptoms in mood and anxiety disorders: results from a nationally representative sample. Sleep.

[34] Boniface S, Kneale J, Shelton N (2014). Drinking pattern is more strongly associated with underreporting of alcohol consumption than sociodemographic factors: evidence from a mixed-methods study. BMC Public Health.

[35] Schuckit MA, Hesselbrock V (2004). Alcohol dependence and anxiety disorders. Focus.

[36] Donker T, Van Straten A, Marks I, Cuijpers P (2011). Quick and easy self-rating of Generalised Anxiety Disorder: validity of the Dutch web-based GAD-7, GAD-2 and GAD-SI. Psychiatry Res.

[37] Kroenke K, Spitzer RL, Williams JBW, Monahan PO, Löwe B (2007). Anxiety disorders in primary care: prevalence, impairment, comorbidity, and detection. Ann Intern Med.

[38] Mossman SA, Luft MJ, Schroeder HK, Varney ST, Fleck DE, Barzman DH, Gilman R, Delbello MP, Strawn JR (2017). The Generalised Anxiety Disorder 7-item scale in adolescents with generalised anxiety disorder: Signal detection and validation. Ann Clin Psychiatry.

[39] Ahn J-K, Kim Y, Choi K-H (2019). The Psychometric Properties and Clinical Utility of the Korean Version of GAD-7 and GAD-2. Front Psychiatry.

[40] Seo JG, Park SP (2015). Validation of the Generalised Anxiety Disorder-7 (GAD-7) and GAD-2 in patients with migraine. J Headache Pain.

[41] Tong X, An D, Mcgonigal A, Park SP, Zhou D (2016). Validation of the Generalised Anxiety Disorder-7 (GAD-7) among Chinese people with epilepsy. Epilepsy Res.

[42] Chan WC, Lam LCW, Chen EYH (2015). Hong Kong: recent development of mental health services. BJPsych Advances.

[43] Kessler RC, Calabrese JR, Farley PA, Gruber MJ, Jewell MA, Katon W, Keck PE, Nierenberg AA, Sampson NA, Shear MK, Shillington AC, Stein MB, Thase ME, Wittchen HU (2013). Composite International Diagnostic Interview screening scales for DSM-IV anxiety and mood disorders. Psychol Med.

[44] Kroenke K, Spitzer RL, Williams JB (2001). The PHQ-9: validity of a brief depression severity measure. J Gen Intern Med.

[45] Zhang YL, Liang W, Chen ZM, Zhang HM, Zhang JH, Weng XQ, Yang SC, Zhang L, Shen LJ, Zhang YL (2013). Validity and reliability of Patient Health Questionnaire-9 and Patient Health Questionnaire-2 to screen for depression among college students in China. Asia Pac Psychiatry.

[46] Angst J, Adolfsson R, Benazzi F, Gamma A, Hantouche E, Meyer TD, Skeppar P, Vieta E, Scott J (2005). The HCL-32: towards a self-assessment tool for hypomanic symptoms in outpatients. J Affect Disord.

[47] Holtmann M, Pörtner F, Duketis E, Flechtner HH, Angst J, Lehmkuhl G (2009). Validation of the Hypomania Checklist (HCL-32) in a nonclinical sample of German adolescents. J Adolescence.

[48] Wu YS, Angst J, Ou CS, Chen HC, Lu RB (2008). Validation of the Chinese version of the hypomania checklist (HCL-32) as an instrument for detecting hypo (mania) in patients with mood disorders. J Affect Disord.

[49] Saunders, J. B., Aasland, O. G., Organisation, W. H. & Others 1987. WHO collaborative project on the identification and treatment of persons with harmful alcohol consumption. Report on phase I: The development of a screening instrument. [Geneva]: World Health Organisation.

[50] Liskola J, Haravuori H, Lindberg N, Niemelä S, Karlsson L, Kiviruusu O, Marttunen M (2018). AUDIT and AUDIT-C as screening instruments for alcohol problem use in adolescents. Drug Alcohol Depend.

[51] Raine A, Benishay D (1995). The SPQ-B: A Brief Screening Instrument for Schizotypal Personality Disorder. J Pers Disord.

[52] Ma WF, Wu PL, Yang SJ, Cheng KF, Chiu HT, Lane HY (2010). Sensitivity and specificity of the Chinese version of the Schizotypal Personality Questionnaire-Brief for identifying undergraduate students susceptible to psychosis. Int J Nurs Stud.

[53] Axelrod SR, Grilo CM, Sanislow C, McGlashan TH (2001). Schizotypal Personality Questionnaire-Brief: factor structure and convergent validity in inpatient adolescents. J Pers Disord.

[54] Bastien CH, Vallières A, Morin CM (2001). Validation of the Insomnia Severity Index as an outcome measure for insomnia research. Sleep Med.

[55] Morin CM, Belleville G, Bélanger L, Ivers H (2011). The Insomnia Severity Index: psychometric indicators to detect insomnia cases and evaluate treatment response. Sleep.

[56] Chung KF, Kan KK, Yeung WF (2011). Assessing insomnia in adolescents: comparison of Insomnia Severity Index, Athens Insomnia Scale and Sleep Quality Index. Sleep Med.

[57] Cook DA, Beckman TJ (2006). Current concepts in validity and reliability for psychometric instruments: theory and application. Am J Med.

[58] Bentler PM (1990). Comparative fit indexes in structural models. Psychol Bull.

[59] Tucker LR, Lewis C (1973). A reliability coefficient for maximum likelihood factor analysis. Psychometrika.

[60] Browne MW, Cudeck R (1992). Alternative Ways of Assessing Model Fit. Sociol Methods Res.

[61] Joreskog KG, Sorbom D (1982). Recent Developments in Structural Equation Modelling. J Marketing Res.

[62] Fluss R, Faraggi D, Reiser B (2005). Estimation of the Youden Index and its associated cut-off point. Biom J.

[63] Andrich D (1978). A rating formulation for ordered response categories. Psychometrika.

[64] Bond, T. G. & Fox, C. M. (2013) Applying the Rasch model: Fundamental measurement in the human sciences.

[65] Smith AB, Rush R, Fallowfield LJ, Velikova G, Sharpe M (2008). Rasch fit statistics and sample size considerations for polytomous data. BMC Med Res Methodol.

[66] Haro JM, Arbabzadeh-Bouchez S, Brugha TS, De Girolamo G, Guyer ME, Jin R, Lepine JP, Mazzi F, Reneses B, Vilagut G, Sampson NA, Kessler RC (2006). Concordance of the Composite International Diagnostic Interview Version 3.0 (CIDI 3.0) with standardised clinical assessments in the WHO World Mental Health Surveys. Int J Methods Psychiatr Res.

[67] Beard C, Björgvinsson T (2014). Beyond generalised anxiety disorder: psychometric properties of the GAD-7 in a heterogeneous psychiatric sample. J Anxiety Disord.

[68] Kertz S, Bigda-Peyton J, Bjorgvinsson T (2012). Validity of the Generalised Anxiety Disorder-7 Scale in an Acute Psychiatric Sample. Clin Psychol Psychotherapy.

[69] Robinson OC, Smith JA (2010). Investigating the Form and Dynamics of Crisis Episodes in Early Adulthood: The Application of a Composite Qualitative Method. Qual Res Psychol.

[70] Pavlova B, Perlis RH, Alda M, Uher R (2015). Lifetime prevalence of anxiety disorders in people with bipolar disorder: a systematic review and meta-analysis. Lancet Psychiatry.

[71] Rihmer Z, Szádóczky E, Füredi J, Kiss K, Papp Z (2001). Anxiety disorders comorbidity in bipolar I, bipolar II and unipolar major depression: results from a population-based study in Hungary. J Affect Disord.

[72] Fiedorowicz JG, Endicott J, Leon AC, Solomon DA, Keller MB, Coryell WH (2011). Subthreshold hypomanic symptoms in progression from unipolar major depression to bipolar disorder. Am J Psychiatry.

[73] Caldwell TM, Rodgers B, Jorm AF, Christensen H, Jacomb PA, Korten AE, Lynskey MT (2002). Patterns of association between alcohol consumption and symptoms of depression and anxiety in young adults. Addiction.

[74] Essau CA (2003). Comorbidity of anxiety disorders in adolescents. Depress Anxiety.

[75] Melegari MG, Bruni O, Sacco R, Barni D, Sette S, Donfrancesco R (2018). Comorbidity of attention deficit hyperactivity disorder and generalized anxiety disorder in children and adolescents. Psychiatry Res.

[76] O’connor DW, Parslow RA (2009). Different responses to K-10 and CIDI suggest that complex structured psychiatric interviews underestimate rates of mental disorder in old people. Psychol Med.

