# Supplementary material for: Assessing anxiety among adolescents in Hong Kong: psychometric properties and validity of the Generalised Anxiety Disorder-7 (GAD-7) in an epidemiological community sample
Source: BMC Psychiatry. 2022 Nov 14;22:703. doi: 10.1186/s12888-022-04329-9 (PMC9664827; doi:10.1186/s12888-022-04329-9)
Supplement: Supplementary file 1 — Additional file 1. [file 12888_2022_4329_MOESM1_ESM.pdf]

Supplementary materials

S1. Probability curves of the four-category GAD-7 rating scale

```
P      -+-----+-----+-----+-----+-----+-----+-----+-----+-----+-----+-
R  1.0 +                                                             +
O      |                                                             |
B      |                                                            3|
A      |0                                                        33 |
B   .8 + 0                  111                                 33   +
I      |  00             111   111                            33     |
L      |    0          11         11                         3       |
I      |     00      11             11         222          3        |
T   .6 +       0    1                 1      22   222     33         +
Y      |        0  1                   1   22        22  3           |
    .5 +         *1                     122            23            +
O      |        1 00                    211            322           |
F   .4 +       1    0                  2   1          3   2          +
       |      1      0               22     1        3     2         |
R      |    11        0             2        1     33       22       |
E      |   1           00         22          11  3           22     |
S   .2 + 11              00      2              13              2    +
P      |1                  00 222              3311              222 |
O      |                   22*00            333    11               2|
N      |              22222     00000  33333         11111           |
S   .0 +**************333333333333333**0000000000000000000***********+
E      -+-----+-----+-----+-----+-----+-----+-----+-----+-----+-----+-
       -5    -4    -3    -2    -1     0     1     2     3     4     5
        PERSON [MINUS] ITEM MEASURE
```

S2. Output of principal component analysis of the item standardised residuals.

	Eigenvalue	Observed
Total raw variance in observations	17.6313	100.0%
Raw variance explained by measures	10.6313	60.3%
Raw variance explained by persons	8.8735	50.3%
Raw variance explained by items	1.7578	10.0%
Raw unexplained variance (total)	7.0000	39.7%
Unexplained variance in 1st contrast	1.4518	8.2%
Unexplained variance in 2nd contrast	1.3220	7.5%
Unexplained variance in 3rd contrast	1.2357	7.0%
Unexplained variance in 4th contrast	1.1170	6.3%
Unexplained variance in 5th contrast	0.9605	5.4%

S3. Largest standardised residual correlation for local dependence analysis.

Item	Item	Correlation
2	3	.07
4	7	−.27
2	6	−.27
3	6	−.25
4	6	−.22
3	5	−.22
1	3	−.21
1	5	−.20
2	5	−.19
1	7	−.18
1	4	−.18
2	4	−.18
3	7	−.18
5	6	−.16
6	7	−.15
3	4	−.14
2	7	−.14
1	6	−.13
1	2	−.12
5	7	−.08

S4. Posteriori factor analysis of goemin-rotated factor loadings for the two-factor structure.

	Factor 1	Factor 2
1. Feeling nervous, anxious, or on edge	0.515	0.301
2. Not being able to stop or control worrying	0.818*	0.040
3. Worrying too much about different things	0.877*	−0.018
4. Trouble relaxing	0.356	0.475*
5. Being so restless that it's hard to sit still	−0.003	0.851*
6. Becoming easily annoyed or irritable	0.249	0.506
7. Feeling afraid as if something awful might happen	0.281	0.448
CFI	*0.970*	
TLI	*0.921*	
SRMR	*0.013*	
RMSEA	*0.099 (0.089, 0.109)*	

CFI = comparative fit index (CFI), TLI = Tucker-Lewis index (TLI), RMSEA = root mean square error of approximation, and SRMR = standardised root-mean-square residual (SRMR). * $p < .05$
